# Human microbiome science: vision for the future, Bethesda, MD, July 24 to 26, 2013

**DOI:** 10.1186/2049-2618-2-16

**Published:** 2014-07-18

**Authors:** Jacques Ravel, Martin J Blaser, Jonathan Braun, Eric Brown, Frederic D Bushman, Eugene B Chang, Julian Davies, Kathryn G Dewey, Timothy Dinan, Maria Dominguez-Bello, Susan E Erdman, B Brett Finlay, Wendy S Garrett, Gary B Huffnagle, Curtis Huttenhower, Janet Jansson, Ian B Jeffery, Christian Jobin, Alexander Khoruts, Heidi H Kong, Johanna W Lampe, Ruth E Ley, Dan R Littman, Sarkis K Mazmanian, David A Mills, Andrew S Neish, Elaine Petrof, David A Relman, Rosamond Rhodes, Peter J Turnbaugh, Vincent B Young, Rob Knight, Owen White

**Affiliations:** 1 Institute for Genome Sciences, Department of Microbiology and Immunology, University of Maryland School of Medicine, 801 W. Baltimore Street, Baltimore, MD 21201, USA; 2 Department of Microbiology, Human Microbiome Program, New York University Langone Medical Center, 550 First Avenue, Bellevue CD 689, New York, NY 10016, USA; 3 Department of Pathology and Laboratory Medicine, David Geffen School of Medicine at UCLA, Los Angeles, CA 90095, USA; 4 The Michael Smith Laboratories and Department of Microbiology and Immunology, University of British Columbia, Vancouver, BC V6T 1Z4, Canada; 5 Department of Microbiology, Perelman School of Medicine at the University of Pennsylvania, Philadelphia, PA 19104, USA; 6 Knapp Center for Biomedical Discovery, University of Chicago, 900 E. 57th Street, Chicago, IL 60637, USA; 7 Department of Microbiology and Immunology, University of British Columbia, 2350 Health Sciences Mall, Life Sciences Centre, Vancouver BC V6T 1Z3, Canada; 8 Department of Nutrition, University of California, One Shields Avenue, Davis, CA 95616, USA; 9 Department of Psychiatry, GF Unity, Cork University Hospital, Cork, Wilton, Ireland; 10 Division of Comparative Medicine, Massachusetts Institute of Technology, One Massachusetts Avenue, Cambridge, MA 02139, USA; 11 Department of Immunology and Infectious Diseases, Harvard School of Public Health, 677 Huntington Avenue, Boston, MA 02115, USA; 12 Department of Internal Medicine/Infectious Diseases, Immunology University of Michigan Medical School, 1500 W. Medical Center Drive, Ann Arbor, MI 48109, USA; 13 Department of Microbiology, Immunology University of Michigan Medical School, 1500 W. Medical Center Drive, Ann Arbor, MI 48109, USA; 14 Department of Biostatistics, Harvard School of Public Health, 655 Huntington Avenue, Boston MA 02115, USA; 15 Earth Sciences Division, Lawrence Berkeley National Laboratory, 1 Cyclotron Road, Berkeley, CA 94720, USA; 16 Department of Microbiology, The Alimentary Pharmabiotic Centre, University College Cork, Cork, Ireland; 17 Department of Infectious Diseases & Pathology, College of Medicine, University of Florida, 2015 SW 16th Avenue, PO Box 110880, Gainesville, FL 32611, USA; 18 Department of Medicine, Division of Gastroenterology, Hepatology & Nutrition, University of Florida, 2015 SW 16th Avenue, PO Box 110880, Gainesville, FL 32611, USA; 19 Department of Medicine, Center for Immunology, Room 3-184, Medical Biosciences Building, 2101 6th S. E, Minneapolis, MN 55416, USA; 20 Dermatology Branch, Center for Cancer Research, National Cancer Institute, National Institutes of Health, 10 Center Dr, Bethesda, MD 20814, USA; 21 Cancer Prevention Program, Public Health Sciences Division, Fred Hutchinson Cancer Research Center, 1100 Fairview Ave N, PO Box 19024, Seattle, WA 98109, USA; 22 Department of Microbiology, Cornell University, 123 Wing Drive, Ithaca, NY 14853, USA; 23 Department of Pathology, Molecular Pathogenesis, 540 First Avenue, Skirball Institute, New York, NY 10016, USA; 24 Department of Microbiology, Molecular Pathogenesis, 540 First Avenue, Skirball Institute, New York, NY 10016, USA; 25 Division of Biology & Biological Engineering, California Institute of Technology, 1200 E. California Bl, Pasadena, CA 91125, USA; 26 Department of Food Science and Technology, University of California, One Shields Avenue, Davis, CA 95616, USA; 27 Department of Viticulture and Enology, University of California, One Shields Avenue, Davis, CA 95616, USA; 28 Department of pathology, Emory University School of Medicine, 105H whitehead bldg., 615 Francis Street, Atlanta, GA 30322, USA; 29 Department of Medicine/Infectious Diseases, Gastrointestinal Diseases Research Unit, Queens University and Kingston General Hospital, 76 Stuart Street, GIDRU wing, Kingston ON K7L 2V7, Canada; 30 Department of Microbiology & Immunology, Stanford University, Stanford, CA 94305, USA; 31 Department of Medicine, Stanford University, Stanford, CA 94305, USA; 32 Department of Medical Education, Icahn School of Medicine at Mount Sinai, One Gustave Levy Place, Box 1076, Annenberg 12-42, New York, NY 10029, USA; 33 FAS Center for Systems Biology, Harvard University, 52 Oxford St, Cambridge, MA 02138, USA; 34 Department of Chemistry and Biochemistry, Howard Hughes Medical Institute, University of Colorado, 215 UCB, Boulder, CO 80309, USA; 35 Institute for Genome Sciences, Department of Epidemiology and Public Health, University of Maryland School of Medicine, 660 W. Redwood Street, Baltimore, MD 21201, USA

## Abstract

A conference entitled ‘Human microbiome science: Vision for the future’ was organized in Bethesda, MD from July 24 to 26, 2013. The event brought together experts in the field of human microbiome research and aimed at providing a comprehensive overview of the state of microbiome research, but more importantly to identify and discuss gaps, challenges and opportunities in this nascent field. This report summarizes the presentations but also describes what is needed for human microbiome research to move forward and deliver medical translational applications.

## Introduction

Each of us consists of about 40 trillion human cells [1] and about 22,000 human genes [2], but as many as 100 trillion microbial cells [3] (the microbiota) and 2 million microbial genes [4] (the metagenome). Understanding the microbial side of ourselves may therefore be critically important for understanding human biology, including drug responses [5-8], susceptibility to infectious [9] and chronic [10] disease, and perhaps even behavior [11]. Since the inception of the Human Microbiome Project (HMP) in 2007 [4,12], the fundamental understanding of the human microbiome has grown at an ever accelerating pace [13]. Together, the HMP healthy cohort study [14,15], and the many associated studies, which provide more details on methodology, bioinformatics analyses, and additional cohorts, have led to over 350 publications (http://www.ploscollections.org/hmp). This work has set the stage for rapid advances, some with high potential for translational studies, in understanding the mechanisms governing the similarities and differences in the microbes we share, their association with diseases, but more importantly, the functional roles microbiota play in health and disease.

### Meeting goals and objectives

To understand the current state of human microbiome research, and to identify key areas for progress going forward, we held a conference in Bethesda, MD from July 24 to 26, 2013, entitled ‘Human Microbiome Science: Vision for the Future’. This conference, which was supported in part from a grant by NIH to the University of Maryland School of Medicine, together with corporate sponsors including Roche, Qiagen, Illumina, Life Technologies, MoBio, Metabolon, and the BioMed Central journal *Microbiome*, sought to provide an overview of cutting-edge work in NIH-supported microbiome research, and to identify obstacles as well as opportunities for progress in this challenging field of research. The meeting was organized by a trans-NIH working group, including 28 participants (programme staff) from 14 (of the 27) NIH Institutes, Centers and Offices, together with four scientific advisory members funded by the Human Microbiome Project. The meeting was attended by 269 participants (and an additional 250 with webcast) from academia, national labs, a range of government agencies including NIH, Environmental Protection Agency (EPA), US Department of Agriculture (USDA), Food and Drug Administration (FDA), US Agency for International Development (USAID), Office of Science and Technology Policy (OSTP), US Army, National Science Foundation (NSF), and National Aeronautics and Space Administration (NASA), and involved 37 speakers from a broad range of disciplines including microbiology, immunology, medicine, infectious disease, ecology, and computer science. The broad expertise of the organizing committee and the participants underscores the way in which microbes pervade the human body and our environment, and microbiome research may soon pervade the biomedical research enterprise.

Over the course of this 3-day meeting, there were presentations and discussions aimed towards:

– Recognizing that the study of the human microbiome, in disease and in health, is of relevance to the missions of all NIH Institutes and Centers;

– Increasing awareness across all NIH Institutes and Centers of gaps, needs, and challenges faced by the broad microbiome research community to drive future research and investments;

– Facilitating coordination between the NIH Institutes and Centers to promote coherent oversight for policies and approaches that will maximally benefit microbiome-related biomedical research;

– Identifying areas where common resources or partnerships would benefit microbiome-related biomedical research;

– Exploring how NIH and other government funding agencies could collaborate to integrate the microbiome into studies of human health and more broadly into studies of human interactions with their physical and microbial environment;

– Fostering understanding of the current state of microbiome research, and shaping an overall vision for future directions of the field over the next 10 years.

### Overview: the human microbiome project

**Figure 1:**
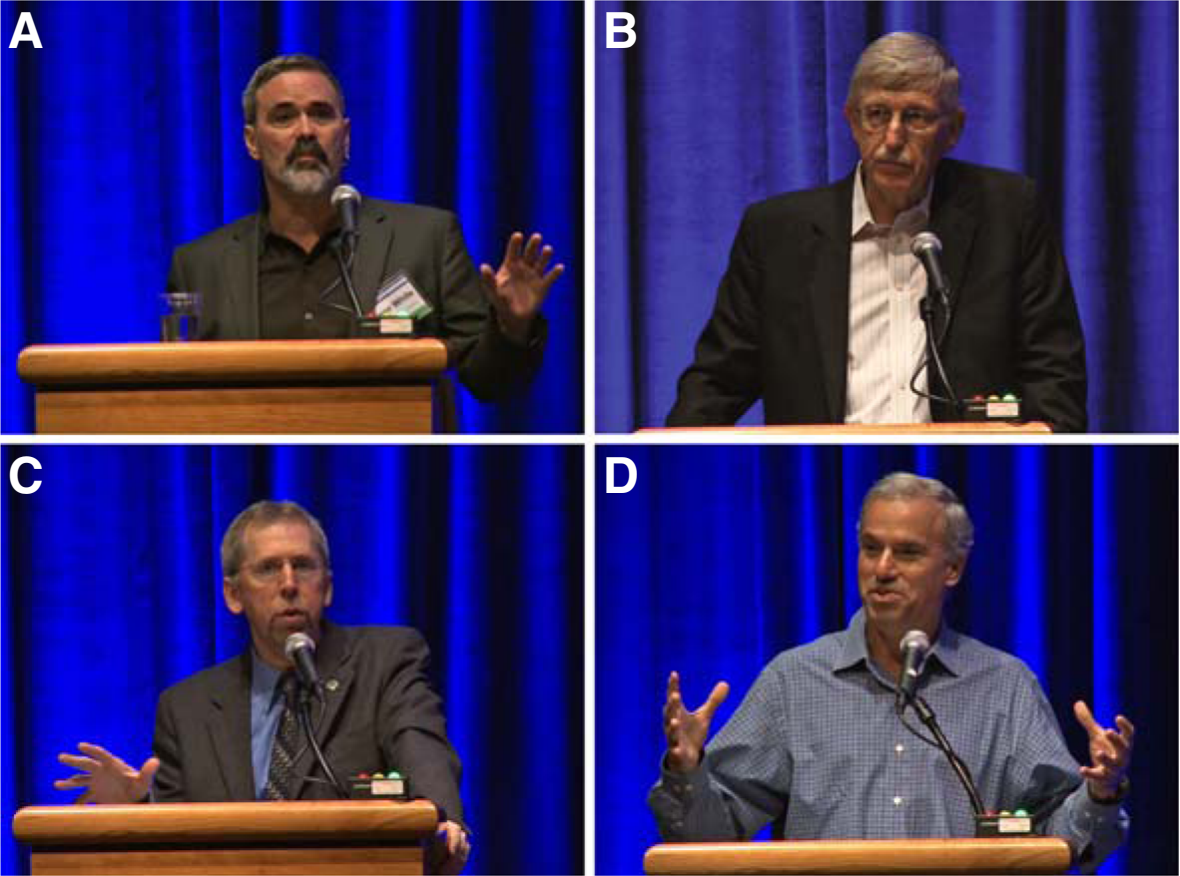
**Dr. Owen White (A), one of the organizers of the meeting introduced Dr. Francis Collins (B), Director of the National Institutes of Health, who gave an historical perspective on the Human Microbiome Project.** Dr. Eric Green **(C)**, Director of the National Human Genome Research Institute discussed the many successes of the Human Microbiome Project. Dr. Jesse Goodman **(D)**, Chief Scientist at the US Food and Drug Administration discussed the regulatory aspects concerning the microbiome, including the challenges associated with fecal transplants.

In the first session Dr. Owen White (University of Maryland School of Medicine, Baltimore, MD, USA) (Figure 1A) set the stage for the conference, noting that the meeting was a unique opportunity for microbiome researchers both to reflect on past successes and to define the direction that the field could take going forward. His introduction was followed by a presentation by Dr. Francis Collins (Director of the National Institutes of Health, Bethesda MD, USA) (Figure 1B), who opened with a historical perspective of the Human Microbiome Project (HMP). He presented an overview of how the microbiome is uniquely positioned to enhance the mission of the NIH because of the many associations between the state of the microbiome and a wide range of diseases from gastrointestinal diseases and conditions, to cancer and even mental illnesses. Dr. Eric Green (Director of National Human Genome Research Institute, NHGRI) (Figure 1C) followed with a report on the state and the many accomplishments of the HMP. The HMP aimed to survey the microbiome in humans through taxonomic and metagenomic analyses. He highlighted the central healthy cohort of volunteers who were intensively sampled, and several demonstration projects that focused on diseases at sites such as the GI track, the skin or the urogenital track. These projects generated over 3.5 Tbp of data and 8 million unique microbial genes were catalogued. These datasets (sequence data, strains, clinical phenotypes, nucleic acid extracts, and even cell lines) are publicly available through repositories and coordinated through a Data Analysis and Coordination Center (DACC) hosted at the Institute for Genome Sciences at the University of Maryland School of Medicine [13]. Overall, the HMP has led to over 350 peer-reviewed scientific publications. The HMP has supported the development of new bioinformatics and technological tools, which altogether facilitate the study of the human microbiome for the scientific community. Human microbiome studies’ ethical, legal and social implications (ELSI) were also evaluated, mirroring the Human Genome Project.

**Figure 2:**
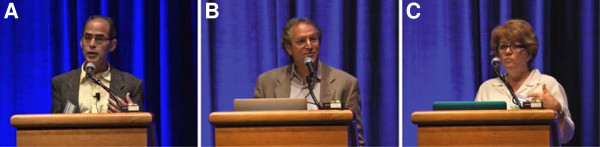
**Keynote speakers.** Dr. Jonathan Braun, University of California Los Angeles, **(A)** highlighted the challenges in translating microbiome sciences. Dr. David Relman, Stanford University, **(B)** discussed the larger role the human microbiome plays in both health and disease. Dr. Maria Dominguez-Bello, New York University, **(C)** discussed aspects of the modern versus ancestral microbiome.

Dr. David Relman (Stanford University) (Figure 2A) gave the first of three keynote addresses on ‘Diversity, Stability and Resilience of the Human Microbiome’, highlighting the larger role the human microbiome plays in both health and disease. He presented recent work from his laboratory on the profound effects of antibiotics in reshaping the human microbiome, and on the value of applying (and perhaps developing) ecological theory for understanding these complex ecosystems and their contributions to human biology [16]. In particular, he raised the issue of resilience, an ecological concept that refers to the amount of disturbance that a system can withstand without changing its self-organizing processes or services [17]. He emphasized the need for better tools to define resilience and evaluate the stability of, and harm to human-associated microbial communities.

Dr. Rosamond Rhodes (Icahn School of Medicine at Mount Sinai, New York) discussed the ethical, legal, and social implications surrounding the study of the human microbiome. In particular, she spoke on issues related to subject identification from knowledge of microbiome sequence data - issues that are in so many ways analogous to those faced by scientists studying the human genome sequence. These issues impact the way microbiome science is and will be performed in the future, especially biobanking. Importantly, they highlight the need to better protect the privacy of subjects involved in microbiome research.

The session was closed by Dr. Robbie Barbero, Office of Science and Technology Policy, (OSTP), who described the role of OSTP in supporting cross-agency collaboration to meet ‘Grand Challenges’ that address important societal needs with a combination of research, technology, and policy inputs. He described several successful Grand Challenges to date, and asked the microbiome community to consider whether a microbiome-focused Grand Challenge might be appropriate at this point in time, especially given the pervasive impact of microbes in the environment and in our bodies.

### Basic biology of the microbiome

This session consisted of three exciting talks describing what we know about how the microbiome develops and changes over time, elaborating on the theme developed earlier by Dr. David Relman. Dr. Ruth Ley (Cornell University) described the changes in the gut microbiota throughout pregnancy, as well as those experienced by newborns in the first few years of life [18]. She presented exciting new work that aims at linking host genetic variations and the composition of the human microbiome. For example, in a cohort of more than 1,000 twin adults, heritable genera of Bacteria and Archaea were identified. Understanding the contribution of human genetic varibility in shaping the composition of the human microbiome will govern the way we will be able to manipulate these microbial communities to maintain or restore health and cure diseases.

Dr. Jacques Ravel (Institute for Genome Sciences, University of Maryland School of Medicine) discussed the ecological principles that govern the dynamics of the human microbiome (resilience, resistance and persistence) and how one can gain better understanding of these dynamic systems using descriptive microbial community compositional surveys [19], gene composition and whole community gene expression or even metabolite analysis. Each of these analyses often reveals different intrinsic dynamic patterns when applied to the same community. His presentation stressed the pitfalls that could result from *a priori* application of principles that might govern microbial community at a given body site to another site. Discussing the vaginal microbiota he introduced a new concept that the intensity of dynamic changes (that is, frequency and duration of change in microbiota composition) could represent an increased risk for acquisition and transmission of sexually transmitted infections. Dr. Frederic Bushman (University of Pennsylvania) then spoke about his work on understanding the dynamics of the human gut virome (the set of viruses that targets bacteria and humans). Although immense progress has been made in understanding the bacteria that inhabit the human body, especially through 16S rRNA gene sequencing and reference genome approaches, studies of the human virome have lagged behind. Analysis of the composition of the gut virome shows that, at least over 2.5 years, the dynamics of the virome is subject-specific and may be important for predicting health, and that the vast majority of viral diversity in the human gut is still uncharacterized and rapidly evolving [20,21]. These studies suggest that a targeted effort to understand viral diversity may be needed given their likely importance for the gut ecosystem as a whole.

### State of the art microbiome tools, technologies, approaches

Microbiome studies rely on the development of novel and robust technologies, approaches and analytical tools. Dr. Janet Jansson (Lawrence Berkeley National Laboratory) described cutting-edge research that combines multi-omics approaches for in-depth characterization of gut community function in health and disease. Taking examples from her own work and those of environmental scientists, she demonstrated the limitations of 16S rRNA gene-based community survey which often shows high variation between subjects, while functionally the communities are more similar. She advocated for applying metaproteomics combined with metabolomics to gain more accurate insights into the function of the community and often the host as well [22,23]. The addition of these multiple layers of omics data provides substantially greater insight into possible mechanisms, and when joined with time-series data can be critical for understanding which differences are causes and which are effects of processes at other levels. However, she stressed that computational tools for functional assignment and for omics’ data integration are still in their infancy and desperately needed. Dr. Dan Littman (New York University) spoke about approaches for studying the interaction of the host immune system and the microbiome, noting the importance of specific commensals in inducing inflammatory T cell responses [24]. He specifically highlighted an association of *Prevotella copri* colonization with the autoimmune response in rheumatoid arthritis [25,26]. Dr. Curtis Huttenhower (Harvard School of Public Health) described novel bioinformatics tools for reconstructing the biomolecular networks driving emergent phenotypes in the microbiome and their influences on human health [27]. These computational methods provide the initial steps to integrate multi-omic data, generate mechanistic hypotheses, and identify actionable molecular targets for therapy. He also discussed the need for improved study designs to effectively scale microbiome investigations to epidemiological populations, which along with principled methods for meta-analysis will aid in ensuring reproducible translational results.

Dr. Rob Knight (University of Colorado at Boulder) described the challenges in moving from associative studies that link microbes to disease towards studies of causality using either germ-free mice models or epidemiological criteria for causation. He also discussed the challenges of integrating human microbiome datasets that use different methodologies and different subject populations. He discussed the critical aspect of the effect size of each metadata element. For example, because age and body site can have large effects even in studies that use very different methods, it is essential to control for technical variability when examining serial samples from the same patient or sub-site analysis (for example, stool *versus* lumen). He also showed the utility of the HMP dataset [14,15] as a data frame for integrating dynamics of time series datasets such as during infant development and for understanding remission of *Clostridium difficile-*associated disease after fecal microbiota transplantation (FMT). Dr. Owen White (University of Maryland School of Medicine) closed the session by discussing integration of large datasets such as those from Human Microbiome Project into accessible resources such as the HMP DACC, which he leads [13]. He emphasized the need for adoption of standards such as those developed by the Genomic Standards Consortium to enable systematic analysis of these integrated datasets [28]. At present, privacy concerns make it difficult to obtain and harmonize data from different projects, especially because a considerable amount of data resides in dbGAP (the access-controlled Database of Genotypes and Phenotypes). He stressed that open resources will go a long way towards making the controlled-access data more re-usable. Finally, he described OSDF (the Open Science Data Framework); an open-source project that provides methods for accessing large volumes of data on distributed file systems, including cloud computing resources, which are increasingly gaining importance as the volume of data expands.

The first day concluded with an open discussion facilitated by Ed Young, a science writer and blogger, between the day’s speakers and the conference participants. A wide range of topics was covered including: the potential of microbiome data to impact health; the need for improved reusability of the resources generated through investment by NIH and other agencies; and the importance of expanding microbiome studies to understand the functional role of the microbiome in disease. To date, most microbiome studies have mostly been associative (associate one or more organisms with a disease state), and the need for studies that are designed to address causality was also discussed. Many audience members were interested in issues of subject identifiability via the microbiome and how a microbiome can be changed in a specific desired direction; both of these topics are areas of intense scientific interest, however, at present a clearer picture is still emerging.

### The modern *versus* the ancestral microbiome

In the second keynote, Dr. Maria Gloria Dominguez-Bello (New York University) (Figure 2B) spoke about the modern versus ancestral microbiome [29,30]. She described how modern practices including hygiene, antibiotics, and limited exposure to livestock have likely affected the composition of the human microbiome. She showed that people living more ancestral lifestyles, without antibiotics and vaccines, such as a previously uncontacted group of the Yanomamö Amerindian tribe in Venezuela, have a profoundly different microbiome as compared to western peoples. Their microbiomes exhibit high diversity not just in the gut, but at all body sites surveyed. She concluded her presentation with a description of a fascinating project that aims to collect not just biological samples, but environmental samples including household surfaces, water, soil, air, domestic pets and livestock along gradients of Westernization in both South America and Africa. Such studies may be critical for understanding the role of microbes, or their loss, in several so-called ‘Western diseases’ which are a high cost burden on the US health system.

### Host immune system/microbiome interactions

Dr. Sarkis Mazmanian (Caltech) discussed how specific bacterial genes control how some gut bacteria colonize the intestinal tract [31]. Focusing on *Bacteroides* species in mice, he presented data demonstrating that a unique class of microbial polysaccharide utilization loci is responsible for species-specific saturable colonization of the crypt channels in the gut. These commensal colonization factors (ccf) loci in *Bacteroides fragilis* and *Bacteroides vulgatus* enable species-specific physical interactions with the host that mediate stable and resilient gut colonization; ccf mutants are defective in horizontal transmission. These studies stress the importance of species-specific genes that, if absent, could affect the establishment of a healthy microbiome. Dr. Eugene Chang (University of Chicago) discussed the effects of the gut microbiome on host epithelial functions and responses [32,33], focusing on the pouchitis model that has been extremely informative in his HMP demonstration project. Dr. Susan Erdman (Massachusetts Institute of Technology) presented her work on animal models showing that specific microbial exposures affect host hormones, including oxytocin [34], interrelated with host immune cell functioning. She showed that prior exposures to gut microbes alter the immune system and potency of T regulatory (Treg) lymphocytes, lowering risk for systemic diseases including cancer later in life. These studies highlighted microbe-endocrine-immune linkages and possible mechanisms for transmitting the effects of maternal microbial exposures to offspring, including behavioral benefits such as improved social interactions.

### Microbiome and disease associations

Dr. Heidi H Kong (NIH, NCI, Dermatology Branch) discussed how skin microbiota can influence host skin immunity and vice versa. For example, Yasmine Belkaid and co-workers [35] showed that applying the skin commensal *S. epidermidis* on germ-free mice can restore the ability to control skin infections by the parasite *Leishmania major*. These findings have potential implications for the development of rational tissue-specific adjuvant and vaccine approaches. In addition to highlighting the importance of studying fungal communities as well as bacterial communities, she discussed work demonstrating the alterations of the skin microbiome in patients with atopic dermatitis (AD) and primary immunodeficiencies [36]. The notable differences in the skin microbiomes of these patient populations may reflect how the host can modulate its skin microbiome and potentially elicit episodes of skin disease. Since AD is often linked with asthma and hay fever, understanding the triggers of AD may allow scientists to develop strategies to prevent and treat these other disorders. Dr. Gary Huffnagle (University of Michigan) described the major challenges associated with sampling the lungs, a body site previously believed to be sterile [37,38]. It is now known that a low-abundance microbiota exists in the lung and that when diseased, the microbial load increases, comprising numerous taxa not found in the mouth or throat. This indicates that there are selective pressures in the lungs for bacterial persistence, colonization and growth that uniquely shape the bacterial community of this body site. Dr. Vincent Young (University of Michigan) reviewed Koch’s Postulates and their implications for moving beyond a ‘classical’ infectious disease model and towards an understanding of the role of both commensal and pathogenic microbes in the development of inflammatory bowel disease. In particular, understanding the interactions of normal gut bacteria with the host mucosa is critical to understanding how dysregulation of these normal interactions can trigger the abnormal host response that characterizes inflammatory bowel disease [32].

### Functional interactions between host and microbiome

Microbes are important modulators of host phenotypes. It is critical to better understand the mechanisms governing this modulation. Dr. Andrew Neish (Emory University) discussed how the microbiome controls epithelial cell proliferation, focusing on the role of microbes in stimulating reactive oxygen species (ROS) production in the gut epithelium [39,40]. In Nox1 and Frp1 null mice, and dNox knockout *Drosophila,* the dynamics of crypt cell proliferation are substantially altered, suggesting that signaling to host cells via reactive oxygen species stimulated by commensal bacteria is important. Dr. Peter Turnbaugh (Harvard University) spoke about the impact of gut microbes on drug metabolism; for example, how certain strains of the gut bacterium *Eggertella lenta* carry a cytochrome operon that causes inactivation of the cardiac drug digoxin [7]. These and other studies highlight the important role of the microbiome in altering the outcome of therapeutics. A better understanding of these interactions may someday allow us to devise strategies to improve drug efficacy and reduce side effects. Dr. Wendy Garrett (Harvard School of Public Health) discussed how microbial metabolites, in particular short-chain fatty acids which are the major end products of bacterial fermentation of dietary polysaccharides, regulate the size and function of the colonic Treg pool, and can protect mice against colitis [41,42]. Thus, short-chain fatty acids may act as a transducer of the gut microbiome into a reliable signal that can regulate immune homeostasis and function in the colon.

### Diet and the microbiome

Dr. Ian Jeffery (University College Cork, Ireland) presented the ElderMet project that studies diet-gut microbiota interactions as it relates to the health of the elderly [43]. In this project, comparisons of elderly subjects living in the community, residential-care or hospital settings were performed using a combination of dietary assessment, gut microbial characterization using 16S rRNA gene surveys, and NMR-based metabolomic analyses. Differences in diet and living situation were highly correlated with differences in the gut microbiota composition and function. Within these populations the microbiota was associated with health outcomes in the individuals such as frailty and inflammatory markers. Over the long term, diet was associated with changes in gut microbiota and therefore dietary modulation of the microbiota may have an impact on health of the elderly. This work could lead to carefully designed dietary interventions to promote healthier aging. Dr. Kathryn Dewey (University of California, Davis) reviewed what is known about the influence of diet in early life on the microbiome. She discussed the key role of breastfeeding, noting that the microbial composition of breast milk may be influenced by the mother’s weight and mode of delivery, and that prebiotics in human milk promote the growth of beneficial gut bacteria. Although many studies have compared breastfed to formula-fed children, it is still unclear which specific aspects of breastfeeding have an effect on the gut microbiome. Introduction of solid foods, the types of solid foods consumed, and certain nutrients such as iron and fatty acids influence the diversity and composition of gut bacteria. However, there is little information on how dietary composition or nutrient intake affects the microbiome of children and the health consequences of differences in the gut microbiome. A new project entitled the Breast Milk, Gut Microbiome, and Immunity (BMMI) Project aims to discover new ways to promote healthy growth in infants and children, and will address some of these important questions. Dr. Johanna Lampe (Fred Hutchinson Research Center and University of Washington) discussed how a range of dietary components are metabolized by bacteria, potentially impacting human health [44]. For example, bacterial metabolites can act as nutrients for host cells (for example, short-chain fatty acids), act as signaling molecules, or be genotoxic (as in the case of nitrites and hydrogen sulfide) or beneficial to host cells (for example, isothiocyanates and flavonoids). Of particular interest in studies of cardiovascular disease is the bacterial conversion of choline to trimethylamine, which is subsequently converted to trimethylamine N-oxide (TMAO) in the liver.

### Translational research and the microbiome

The third day was dedicated to strategies for moving beyond basic association between microbiome and disease by exploiting the microbiome to improve human health. The third keynote was given by Dr. Jonathan Braun (UCLA) (Figure 2C) who noted that the key to translating microbiome science was to identify plausible mechanisms to explain how bacteria might affect the host, as well as therapeutic targets for modulating bacterial activity. He highlighted a number of diseases that were either caused by pathogenic microbes or by a ‘pathogenic’ microbial ecosystem, including *Clostridium difficile*-associated colitis, inflammatory bowel disease, Type 1 diabetes, lymphoma, atherosclerosis, and elements of behavior and cognition. The main challenges are to untangle the relationships among the complex networks of microbial species, their functions and products and how they mediate effects on the host (and vice versa) [45]. Understanding the properties that drive these networks is key to designing reliable interventions. This presentation was followed by Dr. Jesse Goodman (FDA, Chief Scientist) (Figure 1D) who covered regulatory aspects concerning the microbiome, in particular the FDA’s decision to require an IND (Investigational New Drug) application approval for all FMT other than those for treating *C. difficile* infections. In his presentation he also reviewed the regulatory issues surrounding probiotics not covered by the GRAS (Generally Recognized as Safe) guidelines, for example, microbial strains isolated from traditionally consumed fermented foodstuffs. He stressed that the FDA’s mission was to get effective therapies and diagnostics into the hands of consumers as rapidly as possible, however, within the microbiome space methods for demonstrating safety and efficacy are still in their infancy and very much evolving.

### Body/microbiome axis

Dr. Ted Dinan (University College Cork, Ireland) discussed his work on the microbiome-gut-brain axis where he uses germ-free mice to demonstrate that the lack of gut microbes affects sociability, decreases memory, and increases stress responses. He discussed the role that bacteria play in producing neurotransmitters, such as norepinephrine, serotonin, or dopamine, as well as how certain probiotic bacteria can actually modulate the effects of neurotransmitters. In particular, he presented data showing how specific strains of *Lactobacillus rhamnosus* modulate stress and this effect appears to be mediated through the vagus nerve in mice [46]. He cautioned that identifying psychobiotics (a live organism that, when ingested in adequate amounts, produces a health benefit in patients suffering from psychiatric illness) should involve rationally designed screening strategies of very large numbers of putative microbial strains, and that, just as is the case with chemical drugs, most strains are expected to have no effect on most disorders. Dr. Martin Blaser (New York University) spoke about both epidemiological evidence linking antibiotic use and the risk for obesity [47]. He presented experimental evidence in mice that sub-therapeutic antibiotic treatment as well as pulsed full-dose antibiotic treatment modifies body composition, growth, and immune status, leading to increased adiposity. These results, together with the use of antibiotics as growth promoters in livestock, suggest that the obesity epidemic in humans may be in part attributable to modifications of the gut microbiota by antibiotics, especially in early in life. Dr. Stanley Hazen (Cleveland Clinic) discussed links between microbes and cardiovascular disease, and in particular the role of bacteria in converting choline to TMAO, which in turn promotes atherosclerosis [48]. This model, supported by careful work in mice and in human subjects, suggests a direct and specific mechanistic link between gut microbes and cardiovascular disease. Dr. Christian Jobin (University of Florida School of Medicine) presented his work on gut microbiome and colorectal cancer [49], stressing the importance of mechanistic studies identifying specific bacterial genes involved in the causal pathway to cancer. Using IL10-/- germ-free mice which develop colitis-associated colorectal cancer after exposure to microbes, he identified the polyketide colibactin, produced by *E. coli*, which appears to play a substantial inflammation-dependent role in colorectal cancer development in mice and humans. These studies have started to untangle the role of inflammation, gut microbiota, and specific bacterial genes in the development of cancer. Dr. Julian Davies (University of British Columbia, Canada) ended the session with a discussion of the wealth of chemical products that microbes produce, and of the importance of mining this wealth using rational genomic approaches for identifying microbial secondary metabolites with therapeutic activities. He noted that the microbiome itself represents a great source of novel and potentially bioactive natural products [50].

### Probiotics, microbiome vaccines, and fecal transplants

The final session turned to methods for directly manipulating the microbiome. Dr. Alexander Khoruts (University of Minnesota) covered the remarkable efficacy (approximately 90% remission rates) of fecal microbiota transplantation (FMT) in curing recurrent *Clostridium difficile* infection (CDI). The potential mechanisms of action are starting to be elucidated and involve ecological, immunological, and metabolic (bile acids) components - all are proposed to lead to *C. difficile* colonization resistance. He noted that many challenges remain before widespread implementation of FMT can become a reality in routine clinical practice. Dr. Elaine Petrof (Queen’s University, Canada) provided a complementary approach for FMT using synthetic stool made of defined communities of bacteria cultured from human feces but grown in the laboratory, rather than samples from donors. She described the successful treatment of two CDI patients using a defined synthetic stool comprising 33 bacterial isolates [51]. This approach holds substantial promise for improving patient and physician acceptability of FMT. Dr. David Mills (University of California, Davis) discussed the role of milk-oriented prebiotics and probiotics in identifying compounds and bacteria that could form the next generation of rationally-designed prebiotics and probiotics to improve and support infant health. He described the rich diversity in oligosaccharides found in human breast milk and noted that the types of glycans found in breast milk help to shape the composition of the infant gut microbiota, specifically different species of *Bifidobacterium*[52]. This information could be used to design synbiotic formulations comprised of specific human milk oligosaccharides and bacteria to drive a healthy infant gut. Ultimately, understanding the co-evolution of milk glycans, the immune system, and gut bacteria in infancy may be critical in improving human health in infants and may be among the first translational models for modulation of the gut microbiota. Finally, Eric Brown (representing the Brett Finlay Lab, University of British Columbia, Vancouver, Canada) spoke about gut microbiota and vaccine efficacy. In developing countries vaccine efficacy is lower and the gut microbiota is different from that in developed countries. He suggested a link between the impact of diet and malnutrition on the composition of the gut microbiota and vaccine efficacy. He highlighted a need for studies to explore ways to manipulate the gut microbiota to improve vaccine response, including probiotics and/or prebiotics. Understanding the basis for vaccine failure in developing countries is a key issue in global public health. Further, developing tests for the maturation of the infant microbiota and immune system, which could be used to improve public health strategies and vaccine efficacy could have a large beneficial effect.

The meeting closed with another floor discussion moderated by Ed Yong, which gave participants a final chance to share their thoughts about the workshop and about the future of human microbiome research. He led discussions on the impact of antibiotics on the gut microbiota and long-term health, as well as on ways to better understand mechanisms of action driving the benefit associated with manipulation of the microbiota.

### Gaps, needs, and challenges: a framework for the future of human microbiome studies

A key objective of this meeting was to identify gaps, needs and challenges specific to each individual research project presented, and the field as a whole. In addition, the meeting organizers aimed to expand microbiome research by including specialists in other disciplines who could benefit from a microbiome focus. Not surprisingly, speaker responses to the questions of gaps, needs, and challenges varied, nevertheless, some themes emerged:

#### ***Causation and the need for prospective longitudinal studies***

A challenge in microbiome research is to move beyond identification of microbiota community structures that correlate with disease states to establishing a causal link between structural changes and the functions of microbiota in disease. Prospective longitudinal studies in humans were recommended to help better understand the drivers of microbiome dynamics with respect disease risk and to develop predictive models of susceptibility that could suggest better health practices (Relman, Ravel). Prospective long-term follow up of intervention trials are also needed to identify the consequences of differences in microbiome structure and function in early life (Blaser, Dewey, Knight). In mice, multi-generational studies that monitor transfer of microbiota to offspring over generations with changes in diet, antibiotics, or environment are important for testing cause/effects relationships between allergic, autoimmune, and behavioral disorders and the microbiome (Mazmanian). Improved clinical phenotyping and clean study design are essential to successful microbiome studies (Chang). Understanding links between host genetics and the microbiome may also require a longitudinal component, as some phenotypes develop only at specific ages (Ley).

#### ***Improved understanding of microbial ecosystems of the human body***

We need more robust knowledge of ecological networks within microbial ecosystems and their stability over time within individuals (Braun). Characterization of microbial succession and colonization, and of the natural history of microbes and the disorders they cause, was noted as a potential means to correct dysbiosis or influence metabolism of drugs (Mazmanian, Ley, Turnbaugh, Chang). Relman raised several key questions, including: which aspects of diversity matter most, and do these aspects mainly occur at the level of organisms, genes, or pathways, or between communities? He argued that understanding the fitness landscape of an individual would improve our knowledge of resilience and contribute to strategies for maintenance and restoration of important ecosystem services. Mazmanian and Knight also noted that understanding specific and successful colonization processes would be critical to improve disease outcomes.

#### ***Host-microbiome signals and interactions***

Presenters noted our poor understanding of the signaling and communication processes between microbiomes and the host. Improved methods and models systems are needed. While recognizing the limitations of animal models, they nevertheless have provided a wealth of information that have led to our current understanding of the role of the microbiome in health and disease and continue to generate novel hypotheses that can be tested in well-designed human studies. Continued support for the development of better animal models is critical to the future of this field. Studies that investigate the mechanisms by which the microbiota influences and is influenced by the immune system (Littman, Garrett, Chang), or how hormones, such as estrogen impact the vaginal ecosystems (Ravel), are desperately needed. Exhaustive identification of small molecule, bioactive metabolites, secreted and cell surface peptides that can influence microbe-to-host interactions, would be highly desirable (Huttenhower, Mills, Jobin).

#### ***Analysis approaches and tools***

Many concerns regarding the lack of tools for the analysis of ’omic datasets being generated by the microbiome community were expressed during the meeting. Researchers need improved methods to perform quantitative measurements of transcripts, proteins, and metabolites (Braun, Ravel). There is also a lack of consensus as to how much diverse ‘omics’ data is needed for robust scientific interpretation (Knight). The need for better methods to integrate large and diverse ‘omics’ datasets was frequently mentioned (Young, Jeffrey, Lampe, Jansson, Huttenhower, Knight) and the sheer volume of information should be considered to be ‘big data’ problem (Jansson). The lack of procedures to integrate multiple omics data types in longitudinal studies was also identified (Knight). The overall need for increased interdisciplinary collaboration to generate and interpret data was noted (Mills, Lampe, Jansson), mainly because of the different expertise needed to analyze such datasets exceeds what any individual lab can do. Presenters pointed out that tools for the analysis, visualization, and manipulation of these large datasets are also needed. Users would like, for example, to ‘make meta’omics analysis as easy as microarray analysis’ (Huttenhower). Specific systems for analysis of metabolomic, genetic, glycomic datasets (Mills), or sequences from low-biomass samples (Kong), and mass spectrometry data (Davies) are also lacking. Many presentations at the meeting thus revolved around the generation of diverse ‘omics’ datasets, and the challenges associated with integrating that information.

#### ***Standards***

Several presenters expressed that more standards are needed in microbiome research. Standardized protocols improve reproducibility of microbiome experiments and ensure translation of results from independent experiments (Braun, Huttenhower, White, Knight). White suggested that software could be developed to improve the uniformity of data submissions to the NCBI Short Read Archive and dbGaP. Further, uniform clinical and laboratory procedures such as sampling methods at different body sites, PCR protocols, and DNA/RNA extraction methods would also improve our ability to compare data from different research projects (Kong). It was also noted that non-standardized diets for model organisms could account for phenotypic differences between experiments (Mazmanian). Presenters also cited the challenges associated with non-standard metadata associated with microbiome work. Most sample information is not in a standardized format (Knight), and standardized clinical description of phenotypes is lacking (Kong). A solution to these metadata issues would be to utilize software systems similar to the PhenX toolkit for describing common clinical data elements [53], in combination with standardized metadata deposition practices being enforced by scientific journals (White). There was a clear need expressed for collection of data and tools in a single accessible site, much as the HMP DACC provided for the Human Microbiome Project.

## Conclusions

The meeting was clearly a success, in that it highlighted the amazing progress in microbiome research funded across a range of NIH Institute and Centers, focusing on a wide array of diseases. Additionally, while the speakers responses to the needs, gaps, and challenges varied, themes focusing on a few key areas emerged: studies of causality (mechanistic studies in model organisms and prospective longitudinal studies), need to integrate more complex omics and phenotype data, and better standardization of methods and data. Overall, the diversity and excellence of talks underscored how much has been done in this field, in just the past 6 years, and the potential for microbiome science to produce a revolution in human health.

## Abbreviations

AD: Atopic Dermatitis; DACC: Data Analysis and Coordination Center; dbGaP: The Database of Genotypes and Phenotypes; ELSI: Ethical, Legal and Social Implications; EPA: Environmental Protection Agency; FDA: Food and Drug Administration; FMT: Fecal Microbiota Transplantation; GRAS: Generally Recognized as Safe; HMP: Human Microbiome Project; NASA: National Aeronautics and Space Administration; NHGRI: National Human Genome Research Institute; NIH: National Institutes of Health; NSF: National Science Foundation; OSTP: Office of Science and Technology Policy; USAID: US Agency for International Development; USDA: US Department of Agriculture.

## Competing interests

The authors declare that they have no competing interests.

## Authors’ contributions

OW, JR, and RK wrote the paper. All authors read, edited and approved the final manuscript.

## References

[B1] Bianconi E Piovesan A Facchin F Beraudi A Casadei R Frabetti F Vitale L Pelleri MC Tassani S Piva F Perez-Amodio S Strippoli P Canaider S An estimation of the number of cells in the human body Annals Human Biol 2013 40 463 471 10.3109/03014460.2013.807878 23829164

[B2] Pertea M Salzberg SL Between a chicken and a grape: estimating the number of human genes Genome Biol 2010 11 206 20441615 10.1186/gb-2010-11-5-206 PMC2898077

[B3] Savage DC Microbial ecology of the gastrointestinal tract Annu Rev Microbiol 1977 31 107 133 334036 10.1146/annurev.mi.31.100177.000543

[B4] Turnbaugh PJ Ley RE Hamady M Fraser-Liggett CM Knight R Gordon JI The human microbiome project Nature 2007 449 804 810 17943116 10.1038/nature06244 PMC3709439

[B5] Mikov M The metabolism of drugs by the gut flora European J Drug Metabol Pharmacokinet 1994 19 201 207 10.1007/BF03188922 7867662

[B6] Jia W Li H Zhao L Nicholson JK Gut microbiota: a potential new territory for drug targeting Nature Rev 2008 7 123 129 10.1038/nrd2505 18239669

[B7] Haiser HJ Gootenberg DB Chatman K Sirasani G Balskus EP Turnbaugh PJ Predicting and manipulating cardiac drug inactivation by the human gut bacterium Eggerthella lenta Science 2013 341 295 298 23869020 10.1126/science.1235872 PMC3736355

[B8] Haiser HJ Turnbaugh PJ Is it time for a metagenomic basis of therapeutics? Science 2012 336 1253 1255 22674325 10.1126/science.1224396

[B9] Taha TE Hoover DR Dallabetta GA Kumwenda NI Mtimavalye LA Yang LP Liomba GN Broadhead RL Chiphangwi JD Miotti PG Bacterial vaginosis and disturbances of vaginal flora: association with increased acquisition of HIV AIDS (London, England) 1998 12 1699 1706 10.1097/00002030-199813000-00019 9764791

[B10] Tang WH Wang Z Levison BS Koeth RA Britt EB Fu X Wu Y Hazen SL Intestinal microbial metabolism of phosphatidylcholine and cardiovascular risk N Engl J Med 2013 368 1575 1584 23614584 10.1056/NEJMoa1109400 PMC3701945

[B11] Lyte M Microbial endocrinology in the microbiome-gut-brain axis: how bacterial production and utilization of neurochemicals influence behavior PLoS Pathogens 2013 9 e1003726 24244158 10.1371/journal.ppat.1003726 PMC3828163

[B12] Group NHW Peterson J Garges S Giovanni M McInnes P Wang L Schloss JA Bonazzi V McEwen JE Wetterstrand KA Deal C Baker CC Di Francesco V Howcroft TK Karp RW Lunsford RD Wellington CR Belachew T Wright M Giblin C David H Mills M Salomon R Mullins C Akolkar B Begg L Davis C Grandison L Humble M Khalsa J The NIH human microbiome project Genome Res 2009 19 2317 2323 19819907 10.1101/gr.096651.109 PMC2792171

[B13] Gevers D Knight R Petrosino JF Huang K McGuire AL Birren BW Nelson KE White O Methe BA Huttenhower C The human microbiome project: a community resource for the healthy human microbiome PLoS Biol 2012 10 e1001377 22904687 10.1371/journal.pbio.1001377 PMC3419203

[B14] Consortium HMP Structure, function and diversity of the healthy human microbiome Nature 2012 486 207 214 22699609 10.1038/nature11234 PMC3564958

[B15] Consortium HMP A framework for human microbiome research Nature 2012 486 215 221 22699610 10.1038/nature11209 PMC3377744

[B16] Dethlefsen L Huse S Sogin ML Relman DA The pervasive effects of an antibiotic on the human gut microbiota, as revealed by deep 16S rRNA sequencing PLoS Biol 2008 6 e280 19018661 10.1371/journal.pbio.0060280 PMC2586385

[B17] Lemon KP Armitage GC Relman DA Fischbach MA Microbiota-targeted therapies: an ecological perspective Sci Transl Med 2012 4 137rv135 10.1126/scitranslmed.3004183 PMC5725196 22674555

[B18] Koren O Goodrich JK Cullender TC Spor A Laitinen K Backhed HK Gonzalez A Werner JJ Angenent LT Knight R Backhed F Isolauri E Salminen S Ley RE Host remodeling of the gut microbiome and metabolic changes during pregnancy Cell 2012 150 470 480 22863002 10.1016/j.cell.2012.07.008 PMC3505857

[B19] Gajer P Brotman RM Bai G Sakamoto J Schutte UM Zhong X Koenig SS Fu L Ma ZS Zhou X Abdo Z Forney LJ Ravel J Temporal dynamics of the human vaginal microbiota Sci Translat Med 2012 4 132ra152 10.1126/scitranslmed.3003605 PMC3722878 22553250

[B20] Minot S Sinha R Chen J Li H Keilbaugh SA Wu GD Lewis JD Bushman FD The human gut virome: inter-individual variation and dynamic response to diet Genome Res 2011 21 1616 1625 21880779 10.1101/gr.122705.111 PMC3202279

[B21] Minot S Bryson A Chehoud C Wu GD Lewis JD Bushman FD Rapid evolution of the human gut virome Proc Natl Acad Sci U S A 2013 110 12450 12455 23836644 10.1073/pnas.1300833110 PMC3725073

[B22] Jansson JK Neufeld JD Moran MA Gilbert JA Omics for understanding microbial functional dynamics Environ Microbiol 2012 14 1 3 21651688 10.1111/j.1462-2920.2011.02518.x

[B23] Lamendella R VerBerkmoes N Jansson JK ‘Omics’ of the mammalian gut–new insights into function Curr Opin Biotechnol 2012 23 491 500 22626866 10.1016/j.copbio.2012.01.016

[B24] Honda K Littman DR The microbiome in infectious disease and inflammation Annu Rev Immunol 2012 30 759 795 22224764 10.1146/annurev-immunol-020711-074937 PMC4426968

[B25] Scher JU Ubeda C Equinda M Khanin R Buischi Y Viale A Lipuma L Attur M Pillinger MH Weissmann G Littman DR Pamer EG Bretz WA Abramson SB Periodontal disease and the oral microbiota in new-onset rheumatoid arthritis Arthritis Rheumatism 2012 64 3083 3094 22576262 10.1002/art.34539 PMC3428472

[B26] Scher JU Sczesnak A Longman RS Segata N Ubeda C Bielski C Rostron T Cerundolo V Pamer EG Abramson SB Huttenhower C Littman DR Expansion of intestinal prevotella copri correlates with enhanced susceptibility to arthritis eLife 2013 2 e01202 24192039 10.7554/eLife.01202 PMC3816614

[B27] Segata N Boernigen D Tickle TL Morgan XC Garrett WS Huttenhower C Computational meta’omics for microbial community studies Mol Syst Biol 2013 9 666 23670539 10.1038/msb.2013.22 PMC4039370

[B28] Yilmaz P Kottmann R Field D Knight R Cole JR Amaral-Zettler L Gilbert JA Karsch-Mizrachi I Johnston A Cochrane G Vaughan R Hunter C Park J Morrison N Rocca-Serra P Sterk P Arumugam M Bailey M Baumgartner L Birren BW Blaser MJ Bonazzi V Booth T Bork P Bushman FD Buttigieg PL Chain PS Charlson E Costello EK Huot-Creasy H Minimum information about a marker gene sequence (MIMARKS) and minimum information about any (x) sequence (MIxS) specifications Nat Biotechnol 2011 29 415 420 21552244 10.1038/nbt.1823 PMC3367316

[B29] Contreras M Costello EK Hidalgo G Magris M Knight R Dominguez-Bello MG The bacterial microbiota in the oral mucosa of rural Amerindians Microbiol 2010 156 3282 3287 10.1099/mic.0.043174-0 20847007

[B30] Blaser MJ Dominguez-Bello MG Contreras M Magris M Hidalgo G Estrada I Gao Z Clemente JC Costello EK Knight R Distinct cutaneous bacterial assemblages in a sampling of South American Amerindians and US residents ISME J 2013 7 85 95 22895161 10.1038/ismej.2012.81 PMC3526177

[B31] Lee SM Donaldson GP Mikulski Z Boyajian S Ley K Mazmanian SK Bacterial colonization factors control specificity and stability of the gut microbiota Nature 2013 501 426 429 23955152 10.1038/nature12447 PMC3893107

[B32] Young VB Raffals LH Huse SM Vital M Dai D Schloss PD Brulc JM Antonopoulos DA Arrieta RL Kwon JH Reddy KG Hubert NA Grim SL Vineis JH Dalal S Morrison HG Eren AM Meyer F Schmidt TM Tiedje JM Chang EB Sogin ML Multiphasic analysis of the temporal development of the distal gut microbiota in patients following ileal pouch anal anastomosis Microbiome 2013 1 9 24451366 10.1186/2049-2618-1-9 PMC3971607

[B33] Devkota S Chang EB Nutrition, microbiomes, and intestinal inflammation Current Opinion Gastroenterol 2013 29 603 607 10.1097/MOG.0b013e328365d38f 24100722

[B34] Poutahidis T Kearney SM Levkovich T Qi P Varian BJ Lakritz JR Ibrahim YM Chatzigiagkos A Alm EJ Erdman SE Microbial symbionts accelerate wound healing via the neuropeptide hormone oxytocin PLoS One 2013 8 e78898 24205344 10.1371/journal.pone.0078898 PMC3813596

[B35] Naik S Bouladoux N Wilhelm C Molloy MJ Salcedo R Kastenmuller W Deming C Quinones M Koo L Conlan S Spencer S Hall JA Dzutsev A Kong H Campbell DJ Trinchieri G Segre JA Belkaid Y Compartmentalized control of skin immunity by resident commensals Science 2012 337 1115 1119 22837383 10.1126/science.1225152 PMC3513834

[B36] Oh J Freeman AF Program NCS Park M Sokolic R Candotti F Holland SM Segre JA Kong HH The altered landscape of the human skin microbiome in patients with primary immunodeficiencies Genome Res 2013 23 2103 2114 24170601 10.1101/gr.159467.113 PMC3847779

[B37] Dickson RP Huang YJ Martinez FJ Huffnagle GB The lung microbiome and viral-induced exacerbations of chronic obstructive pulmonary disease: new observations, novel approaches Am J Respir Critical Med 2013 188 1185 1186 10.1164/rccm.201309-1573ED 24236585

[B38] Twigg HL 3rd Morris A Ghedin E Curtis JL Huffnagle GB Crothers K Campbell TB Flores SC Fontenot AP Beck JM Huang L Lynch S Knox KS Weinstock G Lung HIVMP: Use of bronchoalveolar lavage to assess the respiratory microbiome: signal in the noise Lancet Respir Med 2013 1 354 356 24429191 10.1016/S2213-2600(13)70117-6

[B39] Neish AS Redox signaling mediated by the gut microbiota Free Radical Res 2013 47 950 957 23937589 10.3109/10715762.2013.833331 PMC5131718

[B40] Jones RM Luo L Ardita CS Richardson AN Kwon YM Mercante JW Alam A Gates CL Wu H Swanson PA Lambeth JD Denning PW Neish AS Symbiotic lactobacilli stimulate gut epithelial proliferation via Nox-mediated generation of reactive oxygen species EMBO J 2013 32 3017 3028 24141879 10.1038/emboj.2013.224 PMC3844951

[B41] Rooks MG Veiga P Wardwell-Scott LH Tickle T Segata N Michaud M Gallini CA Beal C van Hylckama-Vlieg JE Ballal SA Morgan XC Glickman JN Gevers D Huttenhower C 2014 Gut microbiome composition and function in experimental colitis during active disease and treatment-induced remission. ISME J: Garrett WS doi:10.1038/ismej.2014.3 10.1038/ismej.2014.3 PMC4069400 24500617

[B42] Veiga P Gallini CA Beal C Michaud M Delaney ML DuBois A Khlebnikov A van Hylckama Vlieg JE, Punit S, Glickman JN, Onderdonk A, Glimcher LH, Garrett WS: Bifidobacterium animalis subsp. lactis fermented milk product reduces inflammation by altering a niche for colitogenic microbes Proc Natl Acad Sci U S A 2010 107 18132 18137 20921388 10.1073/pnas.1011737107 PMC2964251

[B43] Cusack S O’Toole PW Consortium E Challenges and implications for biomedical research and intervention studies in older populations: insights from the ELDERMET study Gerontology 2013 59 114 121 23146954 10.1159/000343158

[B44] Hullar MA Burnett-Hartman AN Lampe JW Gut microbes, diet, and cancer Cancer Treat Res 2014 159 377 399 24114492 10.1007/978-3-642-38007-5_22 PMC4121395

[B45] McHardy IH Goudarzi M Tong M Ruegger PM Schwager E Weger JR Graeber TG Sonnenburg JL Horvath S Huttenhower C McGovern DP Fornace AJ Jr Borneman J Braun J Integrative analysis of the microbiome and metabolome of the human intestinal mucosal surface reveals exquisite inter-relationships Microbiome 2013 1 17 24450808 10.1186/2049-2618-1-17 PMC3971612

[B46] Cryan JF Dinan TG Mind-altering microorganisms: the impact of the gut microbiota on brain and behaviour Nat Rev Neurosci 2012 13 701 712 22968153 10.1038/nrn3346

[B47] Trasande L Blustein J Liu M Corwin E Cox LM Blaser MJ Infant antibiotic exposures and early-life body mass Int J Obes 2013 37 16 23 10.1038/ijo.2012.132 PMC3798029 22907693

[B48] Koeth RA Wang Z Levison BS Buffa JA Org E Sheehy BT Britt EB Fu X Wu Y Li L Smith JD DiDonato JA Chen J Li H Wu GD Lewis JD Warrier M Brown JM Krauss RM Tang WH Bushman FD Lusis AJ Hazen SL Intestinal microbiota metabolism of L-carnitine, a nutrient in red meat, promotes atherosclerosis Nat Med 2013 19 576 585 23563705 10.1038/nm.3145 PMC3650111

[B49] Arthur JC Jobin C The complex interplay between inflammation, the microbiota and colorectal cancer Gut Microbes 2013 4 253 258 23549517 10.4161/gmic.24220 PMC3669172

[B50] Davies J Ryan KS Introducing the parvome: bioactive compounds in the microbial world ACS Chem Biol 2012 7 252 259 22074935 10.1021/cb200337h

[B51] Petrof EO Gloor GB Vanner SJ Weese SJ Carter D Daigneault MC Brown EM Schroeter K Allen-Vercoe E Stool substitute transplant therapy for the eradication of clostridium difficile infection: ‘RePOOPulating’ the gut Microbiome 2013 1 3 24467987 10.1186/2049-2618-1-3 PMC3869191

[B52] Ruiz-Moyano S Totten SM Garrido DA Smilowitz JT German JB Lebrilla CB Mills DA Variation in consumption of human milk oligosaccharides by infant gut-associated strains of bifidobacterium breve Appl Environ Microbiol 2013 79 6040 6049 23892749 10.1128/AEM.01843-13 PMC3811376

[B53] Hendershot T Pan H Haines J Harlan WR Junkins HA Ramos EM Hamilton CM Haines JL Using the PhenX Toolkit to Add Standard Measures to a Study Current Protocols in Human Genetics 2011 New York: Wiley Chapter 1:Unit1.21 10.1002/0471142905.hg0121s71 21975939

